# Considerations for the Use of AAV‐based Gene Therapy in HIV‐Positive Individuals With Haemophilia

**DOI:** 10.1111/hae.70299

**Published:** 2026-04-25

**Authors:** Jürgen K. Rockstroh, Julia C. Stingl, Marco Stadler, Anja Reichert, Heiner Wedemeyer, Johannes Oldenburg

**Affiliations:** ^1^ University Hospital Bonn Bonn Germany; ^2^ Heidelberg University Hospital Heidelberg Germany; ^3^ BioMarin Europe Ltd. Dublin Ireland; ^4^ BioMarin Deutschland GmbH Kronberg Germany; ^5^ Department of Gastroenterology, Hepatology, Infectious Diseases and Endocrinology Hannover Medical School Hannover Germany; ^6^ Institute of Experimental Haematology and Transfusion Medicine and Centre for Rare Diseases University Hospital Bonn Bonn Germany

**Keywords:** drug interactions, gene therapy, haemophilia, HIV, liver health, treatment considerations

## Abstract

**Introduction:**

There is a high prevalence of human immunodeficiency virus (HIV) infection among the haemophilia community due to treatment in the 1970s and 1980s with contaminated clotting factor. Lifelong treatment regimens for haemophilia and HIV are burdensome alone and pose a particular challenge for individuals living with both conditions. Adeno‐associated virus (AAV)‐based gene therapy restores endogenous factor expression and offers an alternative to routine prophylaxis for haemophilia that individuals living with haemophilia and HIV infection may find uniquely appealing to alleviate the treatment burden for at least one of their chronic conditions.

**Aim:**

The aim of this article is to provide guidance on clinical practice to health care professionals considering gene therapy as a treatment option for individuals with haemophilia and HIV.

**Methods:**

We compile available safety and efficacy evidence from participants living with HIV who participated in gene therapy trials for haemophilia. Then, based on this evidence, we provide several HIV‐specific considerations for individuals with haemophilia and HIV comorbidity.

**Conclusion:**

As part of a shared decision‐making approach, it is important to not only evaluate if gene therapy is appropriate but also to offer recommendations on what to expect when navigating the treatment journey. The available evidence to date indicates modern antiretroviral therapy (ART) regimens may not cause complications when combined with AAV‐based gene therapies. Accordingly, HIV infection should not be considered a general contraindication for gene therapy to restore factor expression in haemophilia. However, a careful consideration of the individual's life context, especially hepatotoxic drug effects or interactions, is warranted.

## 1 Introduction

### 1.1 Haemophilia A and Haemophilia B

Haemophilia A and B are X‐linked inherited bleeding disorders caused by factor VIII (FVIII) or factor IX (FIX) deficiency, respectively [1]. Both are rare disorders, but haemophilia A is comparatively more common and accounts for approximately 80%–85% of all haemophilia cases. Haemophilia is most frequently inherited through an X chromosome mutation in the *F8* or *F9* gene [1]. Residual clotting factor activity is influenced by the nature of the genetic mutation in the respective gene. Alternatively, acquired haemophilia is a rare autoimmune disorder caused by autoantibodies, most commonly those against FVIII [2]. The symptoms and complications for individuals with haemophilia may vary greatly and can include spontaneous bleeding, excessive bleeding following trauma or surgery, joint bleeds, haemorrhages and musculoskeletal complications [1]. The severity of bleeding risk in haemophilia is linked to the degree of clotting factor deficiency and is classified into severe (<1 IU/dL), moderate (1–5 IU/dL), and mild (5–40 IU/dL) haemophilia [1].

### 1.2 Prophylaxis in Haemophilia

Prophylaxis is the standard of care for individuals with severe haemophilia and some individuals with moderate haemophilia [1]. The goal of prophylaxis is to maintain haemostasis and avoid the advanced joint arthropathy that is caused by long‐term bleeding into joints [3]. Modern prophylaxis treatment can involve the routine use of extended half‐life FVIII or FIX products, antibodies that mimic clotting factor activity, or haemostasis rebalancing drugs [1, 3]. However, there was an extended period where FVIII and FIX concentrates used to treat haemophilia were derived from pooled plasma donors before viral inactivation of blood‐derived products was implemented [4, 5]. As a result, individuals with haemophilia receiving FVIII and FIX concentrates, especially before 1985, were at increased risk of exposure to transfusion‐transmitted viral infection from hepatitis A, B, C and D as well as human immunodeficiency virus (HIV) [4, 5]. The mortality of HIV was greatly reduced in 1996 when HAART (Highly Active Antiviral Therapy) became available [6]. Therefore, today in many countries, a significant proportion of older individuals with haemophilia still have HIV treated by potent antiviral drugs [7].

### 1.3 Gene Therapy for Severe Haemophilia

The US Food and Drug Administration and the European Medicines Agency have approved 3 gene therapies for haemophilia: one for haemophilia A (valoctocogene roxaparvovec) and two for haemophilia B (etranacogene dezaparvovec and fidanacogene elaparvovec) [8]. All 3 gene therapies are intended to be delivered as a one‐time, single‐dose, intravenous infusion [8]. As monogenic disorders, haemophilia A and B are well suited for gene therapy as an alternative to routine prophylaxis [8]. Currently, nonreplicating recombinant adeno‐associated virus (AAV) vectors are the leading approach for gene therapy in haemophilia, and they are used in all 3 approved therapies [8]. These liver‐targeted vectors are intended to form stable extrachromosomal episomes capable of delivering a complementary DNA for FVIII or FIX under regulatory control of a liver‐specific promotor [9]. A common treatment‐related adverse event (AE) reported with AAV‐based gene therapies is liver toxicity. In the weeks to months following gene therapy, an immune response targeting transduced hepatocytes is possible and may result in elevated liver enzymes, especially alanine aminotransferase (ALT) [9, 10]. The immune response appears to be complex and includes capsid‐specific adaptive immune responses to transduced hepatocytes. The nature of the immune response suggests that other pathways such as innate immune stimulation or intracellular inflammatory processes might also be involved [10, 11], but the precise mechanism requires further study. If inflammatory responses are left unchecked, they can lead to permanent loss of transgene expression [9]. Therefore, after gene therapy, liver enzymes need to be monitored carefully, and immunosuppressive drugs such as corticosteroids may be needed to control ALT elevations [10]. To date, there are no documented cases of liver failure following AAV‐mediated gene therapy for haemophilia [12].

### 1.4 Reason for Gene Therapy in Individuals With HIV

Treatment options for individuals with haemophilia have improved over the decades, but haemophilia still represents a significant mental and physical burden to activities of daily living [13]. The need for routine infusions of prophylactic treatment can be burdensome and negatively impact quality of life [14]. Haemophilic arthropathy can result from even a few haemorrhages into a joint that trigger synovitis [3, 15], which can then cause pain, reduced mobility, osteoarthritis, and reduced quality of life [15, 16]. Furthermore, intracranial haemorrhage (ICH) is a severe and life‐threatening complication for individuals living with haemophilia [17]. Collectively, due to the burden of repeated therapy administration, physical limitations, and psychosocial consequences, people living with haemophilia often feel that they are missing out on opportunities for education, occupations of choice, sports, and social activities [9].

Transmission of blood‐borne viruses in the 1970s and early 1980s particularly affected many individuals with haemophilia because of their reliance on transfusions of clotting factors to maintain haemostasis. This increased transmission risk, particularly for HIV, placed an even higher burden on the physical and mental health of individuals with haemophilia [18]. HIV is a retrovirus that targets cluster of differentiation (CD)4+ T cells of the immune system, which are crucial for coordinating the body's defence against infections and maintaining homeostatic responses [19, 20]. Over time, the virus depletes CD4+ T cells and weakens the immune system's ability to function effectively, leading to a progressive decline in the immune response [20]. When left untreated, HIV infection progresses to Acquired Immune Deficiency Syndrome (AIDS), which is characterized by severe immune system damage and increased susceptibility to opportunistic infections and cancers [19, 20].

While there is no cure for HIV, antiretroviral therapy (ART) can provide long‐term control over the infection through suppression of viral replication, thus transforming HIV from a fatal disease to a chronic, manageable condition [21]. While prior ART regimens were associated with hepatoxicity, newer ART regimens are safer and carry lower risk of drug‐drug interactions and liver injury [22, 23, 24]. However, for an individual to achieve the full potential of modern ART regimens for clinical and humanistic outcomes, strict adherence (>95%) to therapy regimens is crucial [23]; this is a concept all too familiar to the haemophilia community. While modern ART regimens have improved half‐lives, many still require a minimum of daily dosing [23, 25]. Furthermore, for individuals with both HIV and haemophilia, the burden of managing 2 long‐term chronic illnesses, each with their own treatment regimens, compounds the challenges of daily life. For many, this increases the importance of achieving a haemophilia‐free mindset that decouples activities of daily living from the need for routine prophylaxis [13, 26]. In this regard, gene therapy for haemophilia should also be offered to those with concomitant HIV.

### 1.5 Experience From Clinical Trials

There are limited data available regarding safety and efficacy of gene therapy in HIV‐positive individuals with haemophilia (Table **1**). This is in part due to restrictive inclusion criteria of the relevant clinical trials. As discussed previously, elevations in liver enzymes are commonly reported with AAV‐based gene therapies, and therefore individuals at risk of liver disease have been excluded. For example, the phase 3 international multicentre trials GENEr8‐1 (NCT03370913) and GENEr8‐2 (NCT03392974) evaluating the safety and efficacy of valoctocogene roxaparvovec in individuals with severe haemophilia A initially permitted enrolment of individuals with HIV [27, 28]. Two participants with HIV enrolled in GENEr8‐1, and 1 participant with HIV enrolled in GENEr8‐2. For 2 of the participants, gene therapy provided endogenous FVIII production, reduced need for FVIII infusions, and was associated with no or only grade 1 elevations in liver enzymes that resolved with prednisone. The third participant experienced grade 3 serious adverse events (AEs) related to hepatocellular injury and elevated liver enzymes [28]. Accordingly, the study protocols were subsequently amended to exclude individuals with HIV from participation in the trials [27, 28]. However, follow‐up analysis comparing details of these cases determined all 3 participants were receiving different ART regimens at enrolment. In particular, the AEs that occurred in the third participant were likely caused by negative interactions between the antiretroviral (ARV) drug efavirenz and valoctocogene roxaparvovec. Efavirenz is a nonnucleoside reverse transcriptase inhibitor (NNRTI) with a high risk of hepatotoxicity that is also influenced by variability in drug exposure due to genetic polymorphism in drug metabolism by CYP2B6 [28, 29]. Importantly, the AEs resolved after the participant's ART regimen was changed to raltegravir, emtricitabine, and tenofovir alafenamide [28].

**TABLE 1:** Summary of outcomes for participants in gene therapy trials with haemophilia and HIV

Trial	*N*	History of HBV and/or HCV^a^	ART	ALT/AST‐related AE	Gene therapy‐derived factor level^b^	Efficacy outcome
GENEr8‐1 [27, 28] (NCT03370913)	P1	Y	Emtricitabine, rilpivirine, and tenofovir disoproxil fumarate	None reported	Mild range	Decreased annualized FVIII infusion rate and treated bleed rate
	P2	Y	Darunavir, dolutegravir, and ritonavir^c^	Grade 1 AST elevation; resolved with CS	Mild range	Decreased annualized FVIII infusion rate
GENEr8‐2 [28] (NCT03392974)	P1	Y	Efavirenz,^d^ lamivudine, and tenofovir disoproxil fumarate	Asymptomatic grade 2 AE of AST elevation; asymptomatic grade 3 SAEs of ALT elevation and hepatocellular injury^e^	Severe range^f^	Not effective
SPK‐801130 (NCT03003533 and NCT03432520)	P1	Y	Emtricitabine, rilpivirine, and tenofovir	None reported	Mild range	Decreased annualized FVIII infusion rate and treated bleed rate
	P2	Y	Abacavir,^g^ dolutegravir, lamivudine	None reported	Mild range	Decreased annualized FVIII infusion rate and treated bleed rate
	P3	Y	Elvitegravir, cobicistat, emtricitabine, tenofovir alafenamide, and darunavir	ALT elevation >1.5 times baseline; ART adjusted in response to persistent ALT elevation	Mild range	Decreased annualized FVIII infusion rate and treated bleed rate
HOPE‐B [30] (NCT03489291 and NCT03569891)	P1–5	Y, 4 participants; N, 1 participant	Not specified	ALT elevation of moderate severity in 1 participant; resolved with CS	Mild or normal range	Decreased annualized FIX use and bleed rate

Abbreviations: AE, adverse event; ALT, alanine aminotransferase; ART, antiretroviral therapy; AST, aspartate aminotransferase; CS, corticosteroid treatment; FVIII, factor VIII; FIX, factor IX; HBV, hepatitis B virus; HCV, hepatitis C virus; HIV, human immunodeficiency virus; N, no; P, participant; SAE, serious AE; Y, yes.

a A history of HBV and/or HCV was defined as positivity for HBV or HCV antibodies or prior history of HBV or HCV infection.

b The factor levels are approximations from published sources at 52 to 56 weeks post‐infusion of the respective gene therapy.

c Ritonavir is a protease inhibitor, and while clinically apparent liver injury from full doses of ritonavir have been reported, the use of ritonavir at the lower booster dose is not clearly linked with liver injury [31].

d Efavirenz is a nonnucleoside reverse transcriptase inhibitor with a high risk of hepatotoxicity [28].

e Asymptomatic grade 2 AEs of ALT elevation, AST elevation, and hepatocellular injury were reported; ALT elevation and hepatocellular injury worsened to grade 3 SAEs. These AEs were resolved following adjustment of P1's ART regimen and were thought to be due to drug interactions of efavirenz and valoctocogene roxaparvovec.

f FVIII levels were undetectable at 52 weeks.

g Abacavir is a nucleoside reverse transcriptase inhibitor that carries increased risk of liver toxicity for individuals who are HLA B*5701 positive [32].

An earlier phase 1/2 trial of an additional investigational AAV‐based gene therapy for haemophilia A (giroctocogene fitelparvovec; NCT03003533 and NCT03432520) also enrolled 3 participants with HIV. All 3 participants expressed endogenous FVIII activity derived from the gene therapy, and only 1 of the 3 participants experienced elevations in ALT levels 1.5 times higher than baseline [33]. Although the potential causes of the ALT elevations are not discussed in detail, the participant's ART regimen was adjusted in response to persistent ALT elevations based on the recommendation of the local specialist [33].

For haemophilia B, individuals with HIV were permitted to enrol in the phase 2b (NCT03489291) and phase 3 (NCT03569891) HOPE‐B trials evaluating the efficacy and safety of the AAV‐based gene therapy etranacogene dezaparvovec [30]. In total, there were 5 participants with HIV enrolled in the trials; all expressed FIX derived from the gene therapy, with only 1 of the 5 participants developing a treatment‐related ALT elevation [30]. However, the authors noted the ALT elevation resolved with corticosteroids and concluded the elevation was in line with expectations for individuals without HIV infection [30].

Although the overall number of participants with HIV enrolled in these trials is limited, results show that AAV‐based gene therapy can be a safe therapeutic option for individuals with HIV who are treated with modern ARV medication and have a viral load below 200 copies/mL.

### 1.6 Factors for Successful Gene Therapy

There are several factors that need to be considered in order to maximize the benefits of gene therapy [9, 34]. All gene therapies for haemophilia are liver‐directed; it is therefore important to evaluate the individual's liver health to ensure safety and efficacy. This includes a careful review for a history of liver diseases such as cirrhosis, advanced fibrosis, malignancy, or active or chronic viral infection [35]. While HIV infection is not an absolute contraindication per se, the individual's ART may need to be reviewed for ARV drugs with a known risk for hepatotoxicity [9, 28, 34]. Accordingly, a multidisciplinary team that includes not only the haemophilia treater but also a hepatologist, HIV specialist, and a clinical pharmacologist in situations of polypharmacy and comedication may be necessary to consider if an individual is a good candidate for gene therapy [34, 35]. Hepatotoxicity must be minimized in order to maximize the benefit derived from gene therapy, as clearly illustrated by the clinical trials. Concerns over drug‐induced liver injury should also be extended beyond ART, as other prescribed and over‐the‐counter drugs can lead to hepatotoxicity, especially in individuals carrying pharmacogenetic polymorphisms that affect ART drugs [36].

While gene therapy offers the opportunity for freedom from the hassles of prophylaxis, potential candidates must be made aware of the requirement for regular blood draws and visits at their respective health centre. For example, there will be a need for routine laboratory monitoring and a potential requirement for immunosuppression due to elevations in liver enzymes, especially ALT, a few weeks to months following gene therapy administration [9, 27, 33, 37]. These inflammatory responses will need to be promptly treated with corticosteroids in order to prevent permanent loss of transgene expression. Whereas the use of corticosteroids in untreated individuals with HIV was associated with an increased risk for infectious complications, this is no longer true in the setting of controlled HIV replication and successful immune reconstitution [38]. Steroids are also commonly used for individuals with HIV to treat immune reconstitution syndromes or as a comedication in tuberculosis therapy [38, 39]. Furthermore, the routine laboratory monitoring serves a dual purpose [9]. Gene therapy is a form of treatment with a delayed onset, and therefore it is important to monitor for expression of the respective FVIII or FIX with an appropriate test to inform decisions regarding discontinuation of prophylaxis [9]. In addition to laboratory monitoring, routine ultrasound and elastography examination of the liver should be performed at least annually [35]. To ensure optimum liver health, candidates for gene therapy should also be encouraged to refrain from alcohol for a minimum of 6 months and consider weight management as appropriate following gene therapy infusion [35].

The presence of inhibitors to coagulation factors was an exclusion criterion for many of the gene therapy trials. This decision was initially based on concerns that the anti‐factor immune response might interfere with the efficacy or safety of the gene therapy. However, there is interest in the potential of gene therapy to induce immune tolerance for the restored coagulation factor, and therefore individuals with inhibitors also became eligible for gene therapy [34]. To date, development of FVIII inhibitors in an individual receiving gene therapy has only been reported in a single case in the phase 3 AFFINE study of giroctocogene fitelparvovec [40].

Finally, the serotype of the respective gene therapy (eg, AAV2, AAV5, AAV6, AAV8) will need to be identified and the individual screened for the presence of neutralizing antibodies that could reduce the efficacy of the gene therapy [9]. Wild‐type AAVs can naturally infect humans throughout their lives and, while the seroprevalence varies geographically, the presence of neutralizing antibodies can range from 30% to 60% in humans [41, 42]. The HOPE‐B trial to evaluate etranacogene dezaparvovec for haemophilia B was the only trial to enrol participants regardless of their anti‐AAV5 antibody status and still demonstrated efficacy in participants with preexisting neutralizing antibodies (titres ≤678) [43]. As a result, a deeper understanding around the role of anti‐AAV immunity and the efficacy of gene therapy is needed in order to expand the therapy to a broader population of individuals [34].

## 2 HIV‐Specific Aspects

### 2.1 Comorbidities in HIV

Haemophilia treaters need to be aware of several unique considerations regarding the treatment of individuals with HIV. First, many risk factors for multiple comorbidities have a higher prevalence in individuals with HIV relative to the general population [44, 45]. This includes higher rates of tobacco and substance use, increased body mass index, viral coinfection (hepatitis B and C virus), HIV‐mediated chronic immune activation, polypharmacy, and even specific ARV drugs [44, 46]. For example, metabolic disorders are more common in individuals with HIV. The prevalence of diabetes and dyslipidaemia is up to 6.3% [44, 47] and 69.4% [44, 46, 47] of individuals, respectively. The prevalence of cardiovascular diseases has increased with time, partly due to an ageing community that benefits from the success of ART [47, 48]. For example, hypertension may affect up to 59.6% of individuals with HIV [44, 46, 47]. Issues of mental health including anxiety and depression are also highly prevalent and may affect up to 60% of individuals [44]. Generally speaking, the influence of risk factors on comorbidities and comedications is linked both to age and duration of the HIV infection [21, 44, 45, 46]. This is in part due to the ART regimen itself. While ART has improved life expectancy, its use increases the risk of certain comorbidities [24, 45, 48], and the forecasted multi‐morbidity for individuals with HIV is anticipated to increase to 70% by 2030 [44].

Individuals living with HIV carry a higher risk of developing liver disease compared to those without HIV infection [44, 46, 49]. For example, metabolic dysfunction‐associated steatotic liver disease (MASLD) is now the most common chronic liver disease among individuals living with HIV [50]. Further, the prevalence of liver disease has increased approximately 10‐fold in the modern ART era and is now a leading cause of mortality and morbidity among individuals with HIV [50]. Both the HIV infection itself and the ART regimen are potential risk factors for not only liver diseases like MASLD but also hepatocellular carcinoma (HCC), which is highly associated with liver inflammation [49, 51]. HCC is both more common and carries a worse prognosis (ie, more aggressive disease course) for individuals with HIV infection [49].

Overall liver health is essential to ensure the safety of individuals receiving gene therapy [9]. It is therefore even more important for haemophilia treaters to be aware that many comorbidities, especially metabolic comorbidities, have the potential to adversely affect liver health and may be more prevalent among individuals with HIV [52]. In addition to comorbidities, the risk of liver damage may be increased by the use of polypharmacy. Hepatotoxicity can be aggravated by drug interactions and hepatotoxic drugs with the risk of drug‐induced liver injury. A personalized treatment approach for highly vulnerable individuals that includes clinical pharmacological analysis of all concomitant medications for potential drug interactions or those with hepatotoxic effects is encouraged. In addition, in situations where dosing and polypharmacy make a personalized drug regimen necessary, pharmacogenetic testing to avoid side effects may be recommended [53].

### 2.2 Drug Interactions in AAV‐based Gene Therapy

A careful review of an individual's ART needs to be performed before considering gene therapy. While available evidence for potential drug‐drug interactions between ART and liver‐directed gene therapy are limited, the combination, especially with newer ART, appears to be well tolerated. ARV drugs introduced over recent years offer greater efficacy with improved safety, especially relative to hepatotoxicity observed with earlier ARVs [54, 55]. Modern ART regimens will undoubtedly be encountered among individuals with haemophilia and HIV; however, clinicians will need to be mindful of identifying those individuals who may still have older ARV drugs associated with higher rates of hepatotoxicity. There are several reviews published on the risk of hepatoxicity associated with ARV drugs [54, 55]. For example, newer ARV drugs demonstrated significant improvement in liver function tests such as ALT and aspartate aminotransferase (AST) compared with older ARVs 1 year after treatment initiation [55]. These improvements are exemplified both at the level of mean changes in ALT and AST levels and the percentage of individuals who experience a grade 2 to 4 elevation in liver enzymes [55, 56, 57]. The importance of avoiding drugs with known hepatotoxic effects for individuals considering gene therapy is best exemplified by the participant in the valoctocogene roxaparvovec GENEr8‐2 clinical trial discussed above [28]. Many participants in gene therapy trials experience mild ALT elevations in the weeks to months following gene therapy [9, 27, 33, 37]. However, that participant's concomitant use of the NNRTI efavirenz, a known hepatotoxic drug, was associated with the development of transaminitis with a magnitude and duration that warranted concern. The elevated ALT did not respond to immunosuppression and only resolved once efavirenz was removed from the participant's ART regimen [28]. This example also emphasizes the importance of the “hub and spoke” model for haemophilia care, which seeks to provide a comprehensive approach to delivering gene therapy between dedicated dosing centres and the referring centre. The model seeks to elevate the importance of patient counselling, clear delineation of responsibilities post‐dosing, and close surveillance with patient‐centric follow‐up [9].

### 2.3 Current Recommendations for First‐line Regimens

For individuals with HIV, ART is recommended regardless of their CD4+ T‐cell count [22]. The ART regimens typically include 1 or 2 nucleoside reverse transcriptase inhibitors (NRTIs) with a second‐generation integrase strand transfer inhibitor (INSTI) or 2 NRTIs combined with the NNRTI doravirine [22]. The European AIDS Clinical Society (EACS) guidelines version 13.0 provides 2 ART categories that include 6 first‐line treatment regimens and several additional alternative regimens that may also include protease inhibitors (PIs) [58]. In addition to these treatment guidelines, they also describe the AEs of common ARVs broken down by drug class (eg, NRTIs, NNRTIs, INSTIs, and PIs) [22]. ARVs are considered to be therapeutic agents with a particularly high potential for drug‐drug interactions [22]. Furthermore, some ART regimens, especially the alternative regimens outlined by EACS, include the use of pharmacokinetic boosters such as cobicistat or ritonavir [58, 59]. Hepatic cytochrome P450 3A (CYP3A) is responsible for the metabolism of some ARVs such as the PI drug class. Cobicistat and ritonavir, which itself is a PI, can be used at subtherapeutic levels to achieve pharmacokinetic boosting through the inhibition of CYP3A. One advantage of cobicistat over ritonavir is that it offers more selective inhibition of CYP3A and improved coformulation with other ARVs [23, 59]. Pharmacokinetic boosting is intended to increase the drug exposure of the ARV, lower pill burden, and reduce the dosing schedule [59], all of which can improve adherence to ART [25], but it can also have unwanted clinical implications. For example, inhibition of CYP3A with cobicistat will also inhibit the clearance of other drugs like glucocorticoids [60]. Consequently, the coadministration of cobicistat and glucocorticoids can significantly increase both the half‐life and systemic concentration of the glucocorticoids to a degree that causes hypothalamic‐pituitary adrenal axis suppression and the development of iatrogenic Cushing's syndrome [61, 62, 63].

**FIGURE 1:**
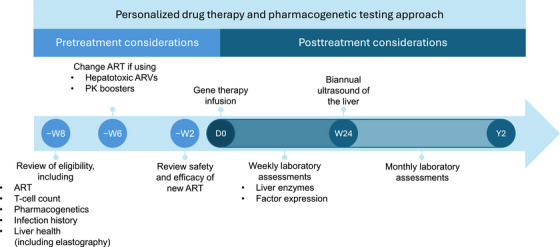
Treatment considerations for individuals with haemophilia and HIV. ART, antiretroviral therapy; ARV, antiretroviral; D, day; HIV, human immunodeficiency virus; PK, pharmacokinetic; W, week; Y, year.

Most of the guidelines recommend first‐line ART regimens that now include the highly potent, unboosted second‐generation INSTIs such as bictegravir and dolutegravir in combination with 1 to 2 NRTIs. All INSTIs except one (elvitegravir) do not require the use of pharmacokinetic boosters, and they have a lower potential for drug‐drug interactions [58, 64, 65]. These attributes, while beneficial in their own regard, may prove especially important for individuals considering gene therapy, as glucocorticoids are often concomitantly administered in the weeks to months following infusion of the gene therapy to control liver inflammation. Therefore, it may be beneficial to switch individuals over to the modern first‐line regimens based on INSTIs and away from those relying on pharmacokinetic boosters prior to the initiation of gene therapy (Figure 1). Individuals with prior virological failure who develop resistance may be on a boosted protease inhibitor‐based salvage therapy. The combination of a second‐generation INSTI together with a newer NNRTI such as doravirine, which is still active even in the presence of a K103N mutation (signature mutation for efavirenz), represent attractive options for managing HIV for these individuals without relying on a booster. However, changing an individual's ART requires careful planning, as switching regimens can be challenging due to the risk of different adverse drug effects and especially if the individual is unwilling or struggling with adherence to therapy. Poor adherence to the new regimen can also cause problems with virologic failure and the development of drug resistance [25]. Accordingly, follow‐up 4 weeks after switching ART is recommended to ensure the safety and efficacy of the new regimen [22] as well as to confirm the viral load is below the detection threshold and that CD4+ T cells are above 200 cells/µL before initiating gene therapy.

### 2.4 Current Recommendations for Viral Coinfections

Careful consideration must also be given to other potential viral coinfections, since some drugs within the HIV‐specific ART regimen (eg, tenofovir disoproxil fumarate [TDF] or tenofovir alafenamide [TAF]) may also be indicated for viral infections like HBV [66]. Especially in these circumstances, the ART should not be changed to TDF/TAF‐free regimens if the individual is hepatitis B surface antigen (HBsAg)‐positive or if the individual has experienced a loss of HBsAg recently.

### 2.5 Recommendations for Follow‐up

After administration of the gene therapy, the follow‐up period for individuals with haemophilia and HIV should be the same as the recommended prescriber guidelines for other individuals. The follow‐up period should focus on controlling immunosuppression with careful monitoring of potential side effects, including changes in blood lipids and glucose, blood pressure, and potential problems with ocular pressure. Haemophilia care providers will need to be well informed on requirements for monitoring individuals with HIV and guidelines on immunosuppression. Generally, it's recommended that individuals with HIV visit their HIV specialist every 3 to 6 months, and during these visits, white blood cell counts and HIV viral load should be determined [67]. However, individuals with HIV may have comorbidities and comedications in addition to the gene therapy that increase the risk of AEs, and thus follow‐up should include personalized drug treatment with patient‐informed safety measures.

## 3 Limitations

The available data on gene therapy for the treatment of haemophilia in individuals with HIV infection is limited to a handful of cases from participants in clinical trials. Further real‐world evidence and long‐term follow‐up is needed to better inform treatment guidance, especially regarding the influence of ART regimens on the efficacy of gene therapy in both haemophilia A and B. In support of this goal, establishing registries will be essential to collect real‐world evidence from the relatively rare but important population of individuals living with haemophilia and HIV. It will also be important for future work and registries to collect data from individuals with antibodies to the hepatitis B core antigen and HIV infection as a distinct group, since best treatment practices in these individuals may require unique considerations.

## 4 Conclusions

We provide several considerations for clinical practice for health care professionals treating individuals with haemophilia and HIV infection with gene therapy. It should be emphasized that HIV infection is not an absolute contraindication for AAV‐based gene therapy, and that individuals with HIV infections have been included in clinical trials. Managing 2 long‐term conditions carries unique challenges and burdens to everyday life. Especially among this community, gene therapy may be perceived as an opportunity. As the combination of AAV‐based gene therapy with especially older HIV medications may increase the risk of drug‐induced hepatoxicity, it is important to monitor for and control hepatoxicity. However, the available evidence suggests that modern ART regimens do not lead to complications from drug interactions when combined with reactive corticosteroids under AAV‐based gene therapy. White blood cell counts, potential drug‐drug interactions, and the impact of prolonged immunosuppression need to be considered when evaluating if AAV‐based gene therapy is appropriate for any individual.

## Author Contributions

All authors contributed to the conceptualization and writing of the manuscript.

## Funding

Medical writing support was funded by BioMarin Pharmaceutical Inc. The concepts presented within the manuscript were originally discussed at an advisory board funded by BioMarin Pharmaceutical Inc.

## Ethics Statement

The authors have nothing to report.

## Conflicts of Interest

Jürgen K. Rockstroh has received payment or honoraria for lectures, presentations, speakers’ bureaus, manuscript writing, or educational events from Abbott, AbbVie, Gilead Sciences, Janssen, MSD, and ViiV Healthcare and honoraria for consulting from Boehringer Ingelheim. Julia C. Stingl has received payment or honoraria for advisory board by BioMarin Europe Ltd. Marco Stadler and Anja Reichert are employees and stockholders of BioMarin Europe Ltd. Heiner Wedemeyer has received grants from AbbVie, Biotest, Gilead, Merck/MSD, and Roche; consulting fees from AbbVie, Aligos, Altimmune, Biotest, BMS, BTG, Dicerna, Enanta, Gilead, Janssen, Merck/MSD, MYR GmbH, Roche, and Vir Biotechnology; and honoraria for speaking from AbbVie, Biotest, Gilead, and Merck/MSD. Johannes Oldenburg received reimbursement for attending symposia/congresses, honoraria for speaking or consulting, or funds for research from Bayer, Biogen Idec, Biotest, Chugai, CSL Behring, Grifols, Novo Nordisk, Octapharma, Pfizer, Roche, Shire, and Sobi.

## Data Availability

The authors have nothing to report.
